# Association between wall metrics and identification of lipid-rich necrotic core in the early-stage atherosclerosis using magnetic resonance imaging

**DOI:** 10.1186/1532-429X-11-S1-P206

**Published:** 2009-01-28

**Authors:** Li Dong, Xue-Qiao Zhao, Baocheng Chu, Hunter Underhill, Thomas S Hatsukami, Chun Yuan

**Affiliations:** Univerisity of Washington, Seattle, WA USA

**Keywords:** Carotid Atherosclerosis, Wall Area, Metrics Measurement, Wall Metrics, Maximum Wall Thickness

## Introduction

Detection of early-stage of carotid atherosclerosis is important for stroke prevention. We sought to explore reliability of wall metrics measurements by high resolution MRI in a population of patients with early-stage carotid atherosclerosis.

## Methods

Subjects (n = 106) recruited from an ongoing clinical trial underwent carotid MRI scans (1.5 T GE scanner) using a high-resolution multi-sequence protocol. Artery metrics measurements, which include maximum wall thickness (MaxWT), mean wall thickness (MWT), normalized wall index (NWI = WA/[LA + WA]), and wall area (WA), were recorded after the lumen and outer wall boundary were identified. Intraclass coefficient (ICC) was used to assess intra- and inter-reader agreements for measurement of the MaxWT, MWT, NWI, and WA. The lipid-rich necrotic core (LRNC) was identified as non-enhancing area on the contrast-enhanced T1-weighted images with exclusion of calcification by multi-sequence images. A spearman correlation coefficient was used to correlate Occurrence of LRNC with artery metrics measurements. Prevalence of LRNC was categorized by NWI in quartiles.

## Results

Intra-reader ICC's for the MaxWT, MWT, NWI, and WA are 0.92, 0.94, 0.95, and 0.92 respectively. Inter-reader ICC's for measurement of the MaxWT, MWT, NWI, and WA are 0.83, 0.80, 0.85, and 0.80 respectively. The LRNC is found in 29 arteries out of a total of 106 arteries (27.4%). The mean area of LRNC is 3.25 mm2 (SD, 2.64). There are significant correlations between occurrence of LRNC and the MaxWT, MWT, NWI, and WA (r = 0.71, 0.66, 0.64 and 0.42, respectively). LRNC is not present in the first NWI quartile and 66.7% of LRNC is present in the last NWI quartile (Table 1).

**Table 1:** Proportion of lipid-rich necrotic core across normalized wall index.

NWI	Number	Proportion	P value (comparing to the 4th quartile)
1st quartile (<= 0.36)	24	0/24 (0%)	< 0.0001
2nd quartile (0.37–0.39)	23	2/23 (8.7%)	0.0001
3rd quartile (0.40–0.45)	32	9/32 (28.1%)	0.007
4th quartile (> 0.45)	23	18/27 (66.7%)	-

## Discussion and conclusion

Small mean LRNC areas found in this study indicate that this study population has early-stage of carotid atherosclerosis. Excellent inter- and intra-reader ICC's suggest that MRI is reliable tool for the evaluation of carotid artery wall metrics. Good correlation between wall metrics measurements and LRNC suggests wall metrics reflect the plaque burden. In particular, NWI can be used to assess prevalence of LRNC in subjects with early-stage carotid atherosclerosis.

